# Supplementary material for: Questionnaires and salivary cortisol to measure stress and depression in mid-pregnancy
Source: PLoS One. 2021 Apr 23;16(4):e0250459. doi: 10.1371/journal.pone.0250459 (PMC8064545; doi:10.1371/journal.pone.0250459)
Supplement: Attachment — Submitted filename: Cortisol_Response to Reviewers_V2.docx [file pone.0250459.s002.docx]

We highly appreciate the Editors’ and Reviewers’ time to review our response and amendments made to the manuscript. We adapted one last section of the manuscript in line with the comment of reviewer #2. The adapted section as well as our response to the comment is described below. The page and line numbers refer to the manuscript version in which the changes are highlighted.

*Reviewer #1: The authors have addressed all of my concerns and I have no remaining comments to the authors. Thank you and congratulations.*

Response: Thank you for this positive remark.

*Reviewer #2: The authors addressed most of my comments. I really acknowledge the modifications made in the introduction section. However, I still have serious doubts about the meaning of reporting a single cortisol level (irrespective of being collected in saliva or plasma) collected during the morning mainly during pregnancy since it will mix the information of the CAR, the global HPA activity and the hypercortisolemia produced during pregnancy.

In any case, I find that the study is interesting and the authors are honest with their results. For these reasons I advise for publication in PLOS one. However, I still have an important consideration to be modified before publication:

1. The authors have added in the revised version of the manuscript “Another potential limitation may be the collection of saliva to measure cortisol levels as marker for HPA activity. Although most studies so far used salivary cortisol as marker of HPA activity, this does not imply that these cortisol levels fully capture HPA activity. Cortisol in blood, hair, or urine may be better markers, but the increased participation burden involved in sampling these materials justifies our choice for salivary cortisol levels as proxy for HPA activity.”. I fully disagree with the statement. Salivary cortisol may be the best marker for HPA activity (definitively better than blodd or spot urine) but the timing of the determination is critical. Evening salivary cortisol provides valuable information about HPA activity whereas sequential collections during the morning provide information about the CAR. Sequential collections during the day provide information about the acrophase and the amplitude of the circadian rhythm of cortisol. The limitation of the describes study is not the use of salivary cortisol but the use of the awakening salivary cortisol as a single point. Please, modify this limitation*

Response: Thank you for this comment, we indeed inadvertently mixed up the limitations. We fully agree with the reviewer that salivary cortisol may the best biomarker for HPA activity, as opposed to blood or urine as suggested, and that timing of cortisol determination is crucial. The limitation of the use of a single cortisol sample was already extensively addressed in the discussion in lines 289-295, but has now been modified and extended as suggested by the Reviewer. The sentences on other markers have been omitted from the manuscript.

Lines: 292-299

[…] having elevated cortisol levels [11]. Timing of cortisol assessment is critical, however. Sequential collection in the morning provides information about the cortisol awakening response, whereas evening salivary cortisol provides information on HPA activity. Although challenging regarding feasibility in large study populations, sequential collection throughout the day provides information on the acrophase and amplitude of the circadian rhythm of cortisol. For feasibility reasons, we chose to collect awakening cortisol levels, as salivary cortisol levels in samples collected immediately after awakening have shown a higher day-to-day correlation compared to afternoon samples [10]. Collecting only a single saliva sample in mid-pregnancy, […]
